# Strongly coupled magnon–phonon dynamics in a single nanomagnet

**DOI:** 10.1038/s41467-019-10545-x

**Published:** 2019-06-14

**Authors:** Cassidy Berk, Mike Jaris, Weigang Yang, Scott Dhuey, Stefano Cabrini, Holger Schmidt

**Affiliations:** 1 School of Engineering, University of California Santa Cruz, 1156 High Street, Santa Cruz, CA 95064 USA; 2 Molecular Foundry, University of California Berkeley, 67 Cyclotron Road, Berkeley, CA 94720 USA

**Keywords:** Ferromagnetism, Magnetic properties and materials, Magneto-optics

## Abstract

Polaritons are widely investigated quasiparticles with fundamental and technological significance due to their unique properties. They have been studied most extensively in semiconductors when photons interact with various elementary excitations. However, other strongly coupled excitations demonstrate similar dynamics. Specifically, when magnon and phonon modes are coupled, a hybridized magnon–phonon quasiparticle can form. Here, we report on the direct observation of coupled magnon–phonon dynamics within a single thin nickel nanomagnet. We develop an analytic description to model the dynamics in two dimensions, enabling us to isolate the parameters influencing the frequency splitting. Furthermore, we demonstrate tuning of the magnon–phonon interaction into the strong coupling regime via the orientation of the applied magnetic field.

## Abstract (web summary)

Exploring the magnon and phonon coupling may enable high efficiency magnonic applications. Here the authors show the observation, understanding and control of the magnon–phonon interaction by studying the magneto-acoustic resonance modes of a single thin-film Ni nanomagnet.

## Introduction

Magnonics is an extremely active research area which exploits the wave nature of magnons, the quanta of spin waves, in order to advance data storage, communication, and information processing technology. However, a current drawback in the excitation, manipulation, and detection of magnons exists due to relatively low conversion efficiencies^1^. Coupling to the phononic system is a less explored avenue for the manipulation of magnons, and has been shown to be a promising means of lowering the switching energy of nanoelements^2,3^. With this in mind, a more thorough understanding of the coupling between the spin and phonon systems in nanostructures is necessary.

Because of this, the magnon–phonon interaction has recently been the focus of increased research, specifically as it is applied to surface acoustic wave ferromagnetic resonance (SAW-FMR)^4–7^. Significant effects of optically excited SAWs on the magnetization dynamics of patterned nanomagnet arrays have been demonstrated^8^. In these systems, the SAW frequencies arise from the geometric arrangement of the elements^9^, with the SAW energy located in the substrate layer below the nanoelements. Due to the magneto-elastic coupling, the magnetic resonance is pinned at the SAW frequency. This effect was used to extract the Gilbert damping parameter in a nanomagnet^10^. In these structures, the dynamics are akin to a driven oscillation rather than to two coupled oscillators^11^. Another exciting development was the experimental demonstration of magnons transferring spin to the phonon system^12^. Despite all of this progress, observation, and quantification of the hybridized magnon–phonon modes itself remain challenging tasks^13^, and so far dynamics of the hybridized modes have not been resolved spectroscopically in relevant structures^14^.

In this article, we utilize the vibrational modes of a single, isolated Ni nanostructure to optically initiate phononic dynamics in the GHz frequency range (5 GHz–25 GHz) along with the intrinsic magnetic resonances of the magnet. Access to this higher frequency range has been a limiting factor in resolving the mode splitting in previous experiments using interdigital transducers^14^. The confined geometry of the magnetic nanostructure isolates the phononic modes much like semiconductor microcavities control excitonic states. Using an external magnetic field in the appropriate geometries, the magnonic mode is tuned through the phononic resonances and the avoided crossings characteristic of coupled systems is observed^15,16^.

## Results

### Hybridized magnon–phonon dynamics

**Fig. 1:**
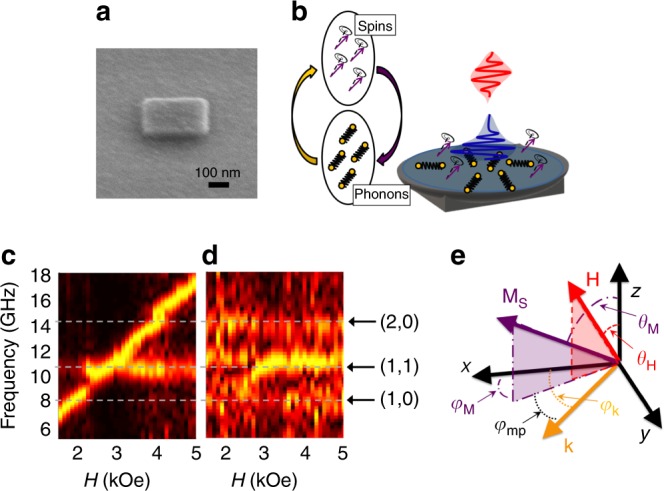
Experimental illustrations and colormaps. **a** Scanning electron microscope image of 330 x 330 x 30 nm Ni nanomagnet. **b** When the pump pulse (400 nm) irradiates the sample, the deposited heat causes the element to thermally expand, which causes the element to vibrate at eigenfrequencies determined by the geometry and material properties. In addition, the heat perturbs the magnetization causing the spins to precess around the effective field. Due to magnetostriction, the spin and phonon systems are coupled to one another. A probe pulse (800 nm) which is delayed in time monitors the dynamics following excitation. **c** Fourier amplitude spectra normalized for each field bin of the magnetic and (**d**) the non-magnetic detection channels. The arrows and dotted lines are indicators of the phononic eigenfrequencies. The positions of these frequencies match in the magnetic and non-magnetic spectra. **e** Experimental geometry. The *x* and *y* axes are defined to be the in-plane directions along the edges of the nanomagnet and the *z*-axis in the direction of the surface normal. The external field **H** is applied at *θ*_H_=60° with respect to the surface normal. This cants the magnetization vector **M**_**S**_ out of the plane to an angle *θ*_M_ with respect to the surface normal and to an in-plane angle, *φ*_*M*_ from the *x*-axis. The phononic modes **k** are characterized by their mode indices and their in-plane angle, *φ*_k_. *φ*_mp_ is the in-plane angle between **M**_**S**_ and **k**

The magnon–phonon dynamics of the nanomagnet (Fig. 1a) were measured using a two-color TR-MOKE setup using a balanced photodiode detection scheme which enables us to measure the magnetic system and the non-magnetic (phononic) system at the same time^17^ (see the Methods section). When the pump pulse hits the nanomagnet, the energy is absorbed by the electron system which then equilibrates with the phonon systems within a few picoseconds according to the two-temperature model^18^. This excites the spin and phonon systems concurrently within the nanomagnet (Fig. 1b).

In Fig. 1, colormaps from the magnetic (c) and non-magnetic (d) channels are displayed. Each field represents a different TR-MOKE scan that has been transformed into the frequency domain using an FFT algorithm. The colors represent the frequency components present at that field. In order to see the frequency variation more clearly, the frequency amplitude was normalized for each field bin. The magnetic channel displays a dominant frequency which changes as the external field changes (Fig. 1c). In addition, there are field independent modes which arise due to the coupling to the phononic system. Confirmation of this is seen in the non-magnetic spectra (Fig. 1d), where the frequencies are all H-field independent and match the positions of the magnetic channel’s field independent frequencies. These spectra look similar to those observed in patterned arrays^8^. However, in the single isolated nanomagnet, the coupling between the two systems is stronger and more direct. It arises within the element itself rather than with surface acoustic waves located in the substrate, leading to qualitatively different behavior around the crossovers between resonances.

The in-plane components of the phononic mode’s k-vector are $k_{x,y} = \frac{{n_{x,y}\pi }}{{l_{x,y}}}$, where *l*_*x,y*_ is the dimension of the nanoelement along the *x* or *y* directions. Therefore, the phononic mode is characterized by the indices (*n*_*x*_*,n*_*y*_) and the in-plane angle from the *x*-axis, *φ*_k_=tan^−1^(*n*_*y*_/*n*_*x*_) (Fig. 1e). Because the material is elastically isotropic, it can be characterized by the Lamé constants, *λ*=*Ev*/(1+*v*)(1−2*v*) and *μ*=*E*/(2(1+*v*)), where *E* is the Young’s Modulus and *v* is the Poisson ratio^19^. We assume the density of Ni, *ρ* = 8900 kg/m^3^ and *v* = 0.31^20^ and fit *E* to the experimentally measured phononic modes using the relation

1 $$\omega _{{\mathrm{ph}}}^2 = \frac{{\left( {2\lambda + 3\mu } \right)k^2}}{{2\rho }}$$

derived in Supplementary Note 2. The fitted value was $209_{ - 29}^{ + 31}$ GPa, in excellent agreement with the range of values reported in ref. ^21^.

In order to extract the relevant magnetic parameters, an identical magnetic film was grown next to the nanostructures. Its precession resonances were measured at various fields and angles, and *γ* and *M*_S_ were fit to the Kittel formula^22^

2 $$\omega _{\mathrm{M}}^2 = \omega _1\omega _2$$

where $\omega _1 = \gamma \left( { - 4\pi M_{\mathrm{S}}\left( {\cos 2\theta _{\mathrm{M}}} \right) + H\cos \left( {\theta _{\mathrm{H}} - \theta _{\mathrm{M}}} \right)} \right)$, $\omega _2 = \gamma \left( - 4\pi M_{\mathrm{S}}\left( {\cos 2\theta _{\mathrm{M}}} \right) + H\cos \left( {\theta _{\mathrm{H}} - \theta _{\mathrm{M}}} \right) \right)$, *H* is the applied field magnitude, *θ*_M_ and *θ*_H_ are the angles of the magnetization vector and the applied field with respect to the surface normal, *γ* is the gyromagnetic ratio, and *M*_S_ is the saturation magnetization. The fitted frequencies yielded values of $\gamma = 1.98_{ - 0.01}^{ + 0.02} \times 10^7$ rad∙ Oe^−1^∙s^−1^ and $M_{\mathrm{S}} = 203_{ - 16}^{ + 8}$ emu∙cm^−3^.

**Fig. 2:**
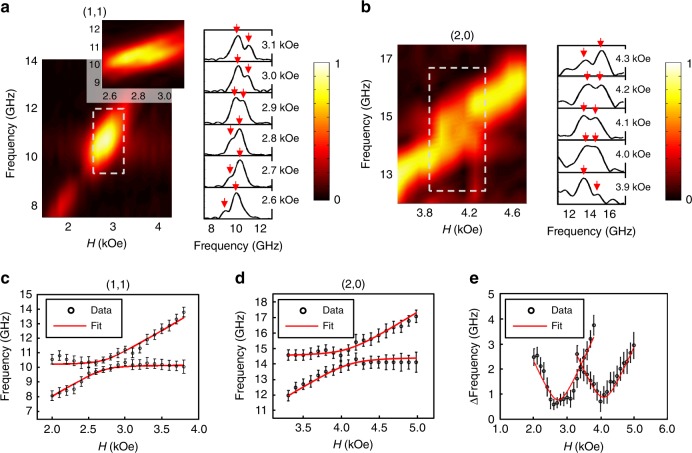
Avoided crossings and fits. Close-ups of the Fourier amplitude spectra exhibiting anti-crossings for the (**a**) (1,1) and (**b**) (2,0) modes. The amplitudes are normalized within each figure. Next to each colormap are the Fourier spectra obtained from the TR-MOKE time trace for the range of applied fields selected by the dotted gray box in the colormap. The two peaks are indicated by the red arrows. The inset in (**a**) is the boxed region Fourier transformed over a longer time length to display the two modes more clearly. **c** Simultaneous fits (solid red lines) of Eq. (3) to the frequencies of the (1,1) and (**d**) (2,0) modes. The error in the frequencies is the FFT resolution obtained from the time duration of each frequency component in the signal. **e** Mode splitting energy of the (1,1) and (2,0) crossings

Changing the external field modifies the frequency of the magnons so they can be brought into resonance with the various phononic modes. The crossings are displayed in Fig. 2a, b for the (1,1) and (2,0) crossings, respectively. The Fourier spectra exhibit two clear peaks at each field value at and around the crossing field. These two frequencies are attributed to the hybridization of the magnon and phonon eigenstates. In this region, the modes do not have a specific magnon or phonon character, but rather exist in both states.

In order to quantitatively characterize the coupling evident in the avoided crossings, we derived a closed-form expression for the eigenfrequencies of the coupled magnon–phonon system. The full derivation is shown in Supplementary Note 2 and yields the characteristic Eq.

3 $$\left( {\omega ^2 - \omega _{\mathrm{M}}^2} \right)\left( {\omega ^2 - \omega _{{\mathrm{ph}}}^2} \right) - \omega _{\mathrm{C}}^4 = 0$$

Where

4 $$\omega _{\mathrm{C}}^4 = \left( {\frac{{\gamma M_{\mathrm{S}}}}{\rho }} \right)\left( {\omega _1C_2 + \omega _2C_1} \right)k^2$$

with

5 $$C_1 = \sin ^22\theta _{\mathrm{M}}\Big( {b_1^2\left( {\cos ^2\varphi _{\mathrm{k}}\cos ^2\varphi _{{\mathrm{mp}}} + \sin ^2\varphi _{\mathrm{k}}\sin ^2\varphi _{{\mathrm{mp}}}} \right)} \\ {+ \frac{3}{4}b_1b_2\sin 2\varphi _{\mathrm{k}}\sin 2\varphi _{{\mathrm{mp}}} + \frac{1}{2}b_2^2\sin ^22\varphi _{{\mathrm{mp}}}} \Big)$$

6 $${C}_2 = \sin ^2\theta _{\mathrm{M}}\left( {{\mathrm{b}}_1^2\sin ^22\varphi _{{\mathrm{mp}}} + 2{\mathrm{b}}_2^2\cos ^22\varphi _{{\mathrm{mp}}}} \right)$$

The frequency peaks were selected from the (1,1) and (2,0) modes and fit simultaneously using Eq. (3), which shows an excellent match between data and fits (Fig. 2c, d). The parameters *γ*, *M*_S_, and *E* from the elastic and magnetic fits were used as initial fitting parameters and allowed to vary within their respective errors. Because the nanomagnet is polycrystalline, *b*_1_ and *b*_2_ are equal. In addition, in order to take into consideration the in-plane magnetization distribution within the nanomagnet, as well as the inevitable slight experimental disorientation of the magnetic field, *φ*_mp_ was also allowed to vary. The fitted value of *b*_1_ was 40 ± 4 × 10^−4^ Oe. Using $b_1 = - \frac{{3\lambda _Sc_{44}}}{{M_{\mathrm{S}}}}$, a polycrystalline magnetostriction value of *λ*_S_ = −34 ± 4 × 10^−6^ was obtained^23^, which is in agreement with the bulk value^24^ of −32 × 10^−6^ and in reasonable agreement with the value measured in Ni thin films^21^. Figure 2e shows the mode splitting corresponding to the coupling strength of the hybridized modes. We find for the (1,1) mode $\Delta f_{{\mathrm{min}}} = 0.76_{ - 0.08}^{ + 0.08}$ GHz and for the (2,0) mode $\Delta f_{{\mathrm{min}}} = 0.85_{ - 0.08}^{ + 0.08}$ GHz. The error is the standard deviation found using a Monte Carlo error-propagation scheme by randomly varying the frequencies within the FFT resolution and then fitting them to Eq. (3). The anticrossing is empirical evidence of the coupling between the magnon and phonon systems, and has not been observed in other experiments utilizing acoustic waves as an excitation mechanism^9,14^. In order to determine the coupling regime of the magnon–phonon resonances, we analyze the loss rates of the different systems by employing a least- squares curve-fitting algorithm to the decaying sinusoids in the time-domain signals. The field-dependent loss rate for the magnetic signal can be approximated by *κ*_M_ = *α*_e_*f*, where *f* is the frequency and *α*_*e*_ is the effective damping^25^. $\alpha _{\mathrm{e}} = 0.038_{ - 0.03}^{ + 0.04}$, and was extracted at 4.6 kOe, away from the crossing point, so that the mode is predominantly magnetic in character. This value is consistent with previous measurements we have made on the damping in Ni^9^. The loss rate of the phononic system, κ_P_, is extracted from the non-magnetic signal. Note that the loss rates cannot be taken directly from the linewidth of the FFT spectra since they are artificially broadened due to the finite duration of the signal in the time domain^26^.

The loss rates are $\kappa _{\mathrm{M}} = 0.41_{ - 0.03}^{ + 0.05}$ GHz for the magnetic system at the (1,1) crossover and $\kappa _{\mathrm{M}} = 0.53_{ - 0.04}^{ + 0.05}$ GHz for the magnetic system at the (2,0) crossover. $\kappa _{\mathrm{P}} = 0.31_{ - 0.07}^{ + 0.12}$ GHz for the (1,1) phononic mode and $\kappa _{\mathrm{P}} = 0.47_{ - 0.07}^{ + 0.12}$ GHz for the (2,0) phononic mode. The coupling rate is given by half of the mode splitting, ${\mathrm{\Gamma }}_{\mathrm{c}} = \Delta f_{{\mathrm{min}}}/2$. This allows us to calculate the cooperativity ${C} = {\mathrm{\Gamma }}_{c}^2/\kappa _{\mathrm{M}}\kappa _{\mathrm{P}}$, which for the (1,1) crossing is ${C} = 1.14_{ - 0.30}^{ + 0.48}$ and for the (2,0) crossing is ${C} = 0.74_{ - 0.14}^{ + 0.21}$. Keeping in mind the error, this places these two crossings in the intermediate coupling regime characterized by 1 > *C* > 0.1^27^.

### Tuning the coupling regime

As evidenced through *C*_1_ and *C*_2_, the splitting energy depends on the type of strains present (normal, *b*_1_; shear, *b*_2_) and is a function of *φ*_mp_ and *θ*_M_. In addition, these angular dependencies are weighted by the magnetic energy terms *ω*_1_ and *ω*_2_ which depend on *M*_S_ and *H* and limit the range of experimentally accessible angles. As a demonstration of the dependence on the in-plane angle of the magnetization and the phononic k-vector, we focus on the (2,0) mode which is characterized by normal strains (*b*_2_ = 0). When both strains are present (depending on the respective amounts), the in-plane dependence disappears due to the orthogonallity of the *b*_1_ and *b*_2_ in-plane angular functions.

**Fig. 3:**
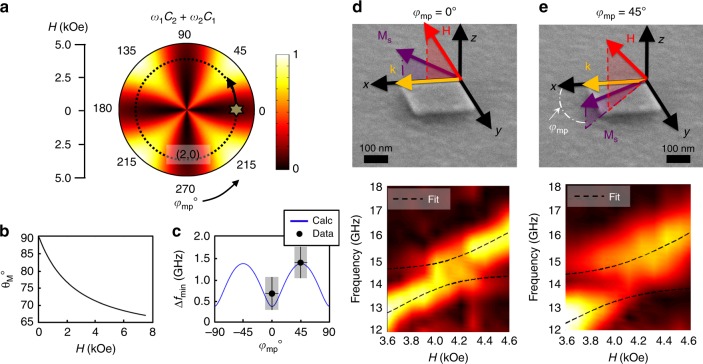
Externally tuning the coupling of the (2,0) mode into the strong coupling regime. **a** Normalized *H* vs. *φ*_mp_ plot of the weighted angular coupling term (*ω*_1_*C*_2_+*ω*_1_*C*_1_). **b** Due to the experimental geometry, only certain out of plane angles of the magnetization *θ*_M_ were accessible for the range of applied fields employed in the experiment. **c** The calculated frequency splitting as a function of *φ*_mp_ (dotted line in **a**) as well as the minimum frequency splitting taken from the data. The y-error was calculated from the FFT resolution. For the x-error a resolution of ±10° was assumed for the in-plane positioning of the nanomagnet. **d** The experimental configuration and the measured spectra with fits to Eq. (3) when the magnetization is oriented along the edge of the square so that it is parallel with the (2,0) phononic mode and (**e**) after rotating the nanoelement so that *φ*_mp_ = 45°. The spectrum shows an increase in the splitting of the two modes and the fit matches the rotation within ±10°

Taking this into consideration, we calculate the weighted coupling strength, (*ω*_1_*C*_2_+*ω*_2_*C*_1_) for our particular experimental configuration and plot it as a function of *H* and *φ*_mp_ (Fig. 3a). The angle of the magnetization, *θ*_M_, from the surface normal is a function of the applied-field configuration. At an applied-field angle of *θ*_H_=60°, Fig. 3b shows the angles of the magnetization *θ*_M_ for the range of applied fields used in the experiment. This *H*-*φ*_mp_ dependence is further modified by the geometric and material properties *ρ*, *γ*, and *M*_S_, which dictate the field at which the two resonances cross. In the original configuration (the applied field along the *x* direction), the (2,0) crossing is shown as a star on the *H*-*φ*_mp_ plot (Fig. 3a). By rotating the nanomagnet in-plane, we can change *φ*_mp_, which changes the coupling strength (moving along the dotted line in Fig. 3a) and is maximum at an angle of 45°. The calculated and experimentally measured minimum frequency splitting versus *φ*_mp_ are shown in Fig. 3c. The data match the calculated results quite well. Figure 3d shows the configuration and obtained spectra with the magnetization oriented parallel to the (2,0) mode, and Fig. 3e shows the case where the magnetization is oriented 45° to the (2,0) mode. For the 45° spectrum, *φ*_mp_ was fit to Eq. (3) using the previously obtained material parameters as constants. The value obtained was *φ*_mp_ = 50°, a very close match. More importantly, from the spectrum in Fig. 3e, it is evident that the splitting increases for *φ*_mp_ = 45° the bandgap starts to emerge in the spectrum. The frequency splitting increases to $\Delta f_{{\mathrm{min}}} = 1.41_{ - 0.16}^{ + 0.16}$ GHz, a 66% increase from the *φ*_mp_ = 0° case. The damping in this configuration was also measured at 4.6 kOe, and is $\alpha _{\mathrm{e}} = 0.047_{ - 0.03}^{ + 0.03}$ which corresponds to $\kappa _{\mathrm{M}} = 0.65_{ - 0.04}^{ + 0.05}$. At this crossing ${\mathrm{\Gamma }}_{\mathrm{c}} > \kappa _{\mathrm{M}}$,*κ*_P_, which translates into a cooperativity of ${C} = 1.65_{ - 0.32}^{ + 0.48}$, placing this crossing in the strong coupling regime within error (*C* > 1). Therefore, by reorienting the magnetization vector using an externally applied magnetic field, we were able to increase the coupling from the intermediate into the strong coupling regime. Table 1 shows the rate values and cooperativities for the three crossings analyzed.

**Table 1:** Coupling, loss rates and cooperativities for the different crossings

Crossing	${\mathrm{\Gamma }}_{\boldsymbol{c}}$ (GHz)	${\mathrm{\kappa }}_{\mathrm{M}}$ (GHz)	${\mathrm{\kappa }}_{\mathrm{P}}$ (GHz)	C
(1,1)	$0.38_{ - 0.04}^{ + 0.04}$	$0.41_{ - 0.03}^{ + 0.05}$	$0.31_{ - 0.07}^{ + 0.12}$	$1.14_{ - 0.30}^{ + 0.48}$
(2,0), 0^o^	$0.43_{ - 0.04}^{ + 0.04}$	$0.53_{ - 0.04}^{ + 0.05}$	$0.47_{ - 0.07}^{ + 0.12}$	$0.74_{ - 0.14}^{ + 0.21}$
(2,0), 45^o^	$0.71_{ - 0.08}^{ + 0.08}$	$0.65_{ - 0.04}^{ + 0.05}$	$0.47_{ - 0.07}^{ + 0.12}$	$1.65_{ - 0.32}^{ + 0.48}$

## Discussion

Hybridized magnon–phonon dynamics were measured by tuning the magnonic resonance to that of the intrinsic phononic vibrations of an isolated square nanomagnet. The hybridized modes were clearly resolved in the Fourier transforms of the time-dependent magneto-optic signals at resonance. A two-dimensional equation describing the dynamics was derived which fit the data with high accuracy. In addition, the coupling was shown to depend on the orientation of the magnetization vector and the phononic vector as predicted by the theory. By tuning the direction of the applied field, we were able to enhance the coupling so that the system was unambiguously in the strong coupling regime. The ability to uniquely tune the energy splitting of the hybridized mode using various external degrees of freedom is attractive from the perspective of reconfigurable magnonic devices^28,29^. In addition, this magnetomechanical system provides a means of studying the dynamics of coupled quantum systems which may aid in the development of more efficient transducers between phononic and magnonic systems.

## Methods

### Sample fabrication

A polycrystalline Ni square with dimensions 330 x 330 x 30 nm was fabricated using electron beam lithography on a (100) Si substrate capped by a 110-nm-thick hafnium oxide antireflection coating for enhanced magneto-optic sensitivity (Supplementary Fig. 1)^23^.

### Experimental setup

The dynamics of this nanomagnet upon pulsed optical excitation were measured using a two-color TR-MOKE setup (Supplementary Fig. 1b). In all, 800 -nm 165-fs pulses exit the femtosecond laser cavity at a repetition rate of 76 MHz. They are split by a beam splitter (BS1), and part of the light is sent through a second harmonic generator (SHG) where the frequency is doubled to produce the pump pulse at a wavelength of 400 nm. The pump pulse is then sent through a mechanical chopper wheel (MCW), which serves as a reference to the lock-in amplifiers. The other component of light serves as the probe pulse and has a much lower power than the pump pulse so that it has a negligible effect on the magnetic system. After the beam splitter, the probe pulse goes through a delay stage to adjust the arrival time Δt of the probe pulse and then through a polarizer (*L*(*α*_*P*_)). The two beams are recombined using a dichroic filter (DF). Both beams are then focused onto the sample using a ×100 microscope objective controlled by a Witec Microscope (WM) which is able to position the objective and the sample with submicron resolution. Due to a slight chromatic aberration from the objective, the two beams are defocused from one another which provides a larger radius of the pump beam at the focal point of the probe beam and ensures a homogeneous excitation of the nanoelement.

The reflected beams are collected by the objective and sent back along their incoming paths. When the probe reaches BS2, it is diverted from the path. A color filter is in place so as to get rid of any residual blue pump energy. The red beam is then split by a polarizing beam splitter (PBS) and focused onto a pair of photodiode detectors (BPD). By adjusting *L*(*α*_*P*_), the signal can be balanced so the photodiodes’ voltages are equal. The detectors are connected to a circuit which outputs the difference (Δ) and sum (Σ) signals of the two photodiodes to two lock-in amplifiers which are then read by a LabView program. Due to the magneto-optic Kerr effect, the polarization of the light is rotated after reflection off the sample. This changes the relative magnitudes of the components of light split by the PBS which affects the difference (magnetic) signal. The sum (non-magnetic) signal is unaffected by changes in polarization, but is affected by changes in the reflectivity which occur due to elastic modulation of the dielectric tensor^30^. Furthermore, since changes in reflectivity affect both components of the light split by the PBS equally, the difference channel is unaffected by reflectivity changes. In this way, the magnetic and elastic signals can be separated and detected simultaneously^17^.

An external magnetic field was applied using a pair of Nd permanent magnets and the orientation and magnitude of the field was characterized using a gauss meter. The magnetic field, **H**, was applied at *θ*_H_ = 60° with respect to the surface normal. This angle was chosen so that the magnonic system could be tuned to multiple phononic resonances.

## Supplementary information

Supplementary Information

## Data Availability

The data that support the findings of this study are available from the corresponding author upon reasonable request.

## Code availability

The code that support the findings of this study are available from the corresponding author upon reasonable request.
