# Supplementary material for: Strongly coupled magnon–phonon dynamics in a single nanomagnet
Source: Nat Commun. 2019 Jun 14;10:2652. doi: 10.1038/s41467-019-10545-x (PMC6570761; doi:10.1038/s41467-019-10545-x)
Supplement: Supplementary file 1 — Supplementary Information [file 41467_2019_10545_MOESM1_ESM.pdf]

Strongly Coupled Magnon-Phonon Dynamics in a Single Nanomagnet Supplementary Information

Cassidy Berk[1],* Mike Jaris[1], Weigang Yang[1], Scott Dhuey[2], Stefano Cabrini[2] and Holger Schmidt[1]

[1]*School of Engineering, University of California Santa Cruz, 1156 High Street, Santa Cruz, California 95064, USA*

[2]*Molecular Foundry. University of California Berkeley, 67 Cyclotron Road, Berkeley, California, 94720, USA*

*Corresponding author: crberk@soe.ucsc.edu

Supplementary Note 1. Experimental Schematic

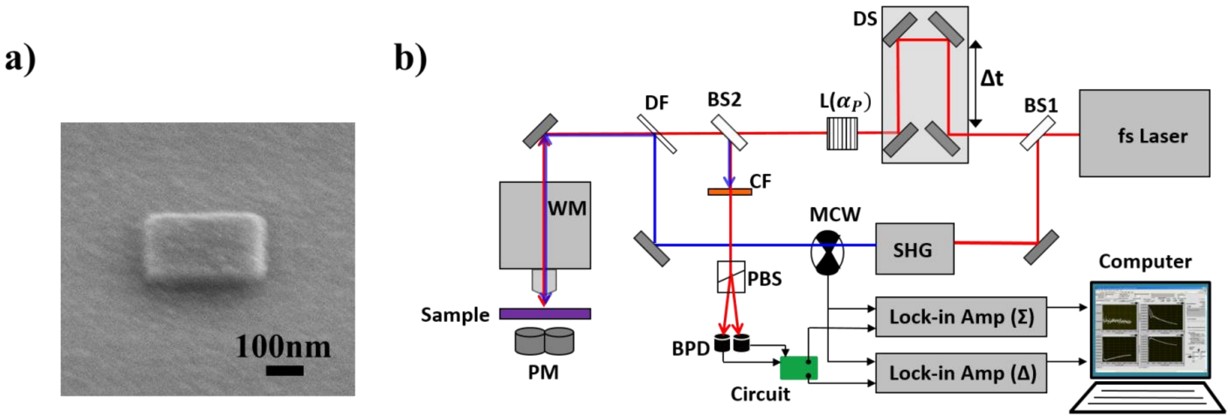

Supplementary Figure 1: (a) Scanning electron microscope image of 330nm x 330nm x 30nm Ni nanomagnet. **(b)** Schematic illustration of the experimental setup described in the text. Beam Splitter (BS); Delay Stage (DS); Linear Polarizer ($L(\alpha_P)$); Dichroic Filter (DF); Witec Microscope (WM); Permanent Magnets (PM); Second Harmonic Generator (SHG); Mechanical Chopper Wheel (MCW); Color Filter (CF); Polarizing Beam Splitter (PBS); Balanced Photodiodes (BPD)

Supplementary Note 2. Analytic Derivations

First, the equations describing the individual phonon and magnon dynamics are introduced. Then the coupling term which is responsible for the hybridized dynamics is considered. The basis for the derivation as well as the symbolic representation has been adopted from Supplementary ref. [1]

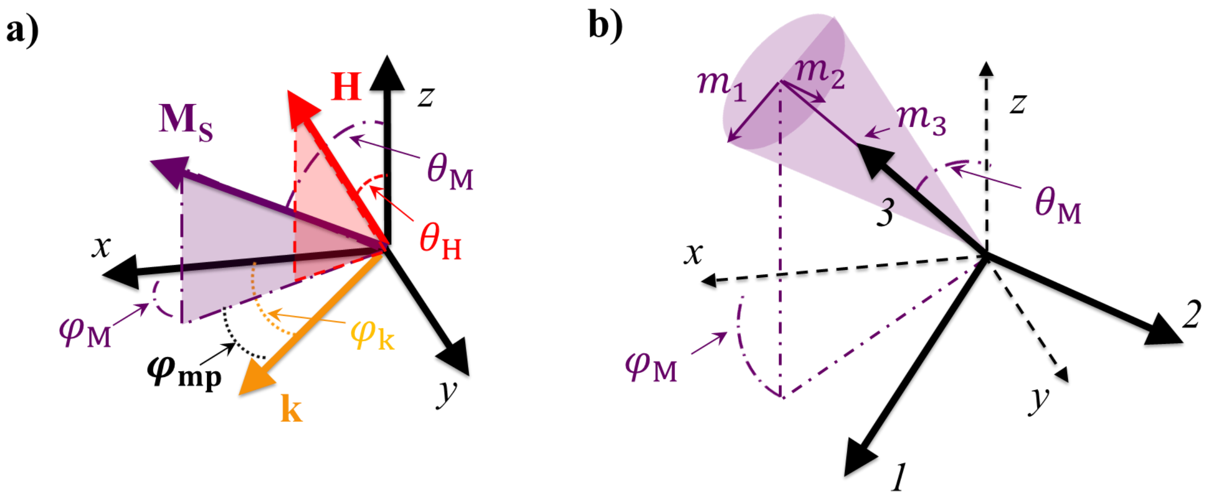

Supplementary Figure 2: (a) In the {*x*,*y*,*z*} coordinate system The *x* and *y* directions are defined by the edges of the nanomagnet and the *z*-direction is the surface normal. The external field **H** is applied at $\theta_H = 60°$ with respect to the surface normal. This cants the magnetization vector $\mathbf{M_S}$ out of the plane to an angle θ_M with respect to the surface normal and to an in-plane angle, φ_M from the x-axis. The phononic modes **k** are characterized by their mode indices and their in-plane angle, φ_k. φ_{mp} is the in-plane angle between $\mathbf{M_S}$ and **k**. **(b)** The Magnetization precession can be mapped to the {*1*,*2*,*3*} coordinate system defined by the direction of the Magnetization vector and the plane of the cone due to the precession of $\mathbf{M_S}$. m_3 is along the direction of $\mathbf{M_S}$ at equilibrium, m_2 lies in the film plane and m_1 is orthogonal to m_2 and m_3.

2d Elastic Dynamics

The elastic energy density is given by:

$$W = \frac{1}{2} C_{ijkl}\varepsilon_{ij}\varepsilon_{kl} \qquad (1)$$

Where $i, j = \{x, y, z\}$, C_{ijkl} are the components of the stiffness tensor, $\varepsilon_{ij} = \left(\frac{\partial u_i}{\partial x_j} + \frac{\partial u_j}{\partial x_i}\right)/2$ are the strain components and u_i is the displacement vector. In order to quantify the phononic eigenmodes the elastic wave equation is solved

$$\rho \frac{\partial^2}{\partial t^2} u_i = \sum_{j=1}^{3} \frac{\partial \sigma_{ji}}{\partial x_j} \tag{2}$$

Where

$$\sigma_{ik} = \frac{\partial W}{\partial \varepsilon_{ik}} \tag{3}$$

is the Cauchy stress tensor and ρ is the density of the material. Due to the small z dimension of the element we can consider the nanomagnet to be two-dimensional in the x and y directions (Supplementary Fig. 2).[2]

We assume a solution of the form $u_{x,y} = u_{x,y}^{0} e^{i(\boldsymbol{k}\cdot\boldsymbol{r} - \omega t)}$ where $k^2 = \sqrt{k_x^2 + k_y^2}$, $k_{x,y} = \frac{n_{x,y}\pi}{l_{x,y}}$ and $l_{x,y}$ is the dimension of the nanoelement along the *x* or *y* direction.[3] Furthermore, since each phononic mode is degenerate for every value of k_x and k_y we set $k_{x,y} = \frac{k}{\sqrt{2}}$ which ensures $k^2 = {k_x}^2 + {k_y}^2$. The in-plane angle of **k** is $\varphi_{\mathrm{k}} = \tan^{-1}(n_y/n_x)$. Because the system is isotropic, the expansion of the element due to a heat pulse from the laser is the same in the *x* and *y* directions such that $u_x^0 = u_y^0$. The system of equations can be solved by setting the Determinant equal to zero. This yields the eigenfrequencies for the phononic system given by

$$ {\omega_{ph}}^2 = \frac{(2\lambda + 3\mu)k^2}{2\rho} \tag{4}$$

Where the Lame parameters μ and λ are given by $C_{xxxx} = 2\mu + \lambda, C_{xxyy} = \lambda$ and $C_{xyxy} = \mu$. The Lame parameters can be related to the Young's Modulus, E and the Poisson ratio, ν through

$\lambda = E\nu/(1+\nu)(1-2\nu)$ and $\mu = E/\big(2(1+\nu)\big)$.[4] Assuming the density of Ni, ρ = 8900 $\mathrm{kg/m^3}$ and ν = 0.31,[5] the experimentally measured phononic mode frequencies were fit to Supplementary Equation (4) (Supplementary Fig. 3) yielding a value for the Young's Modulus of 209^{+31}_{-29} GPa which matches well with literature values.[5]

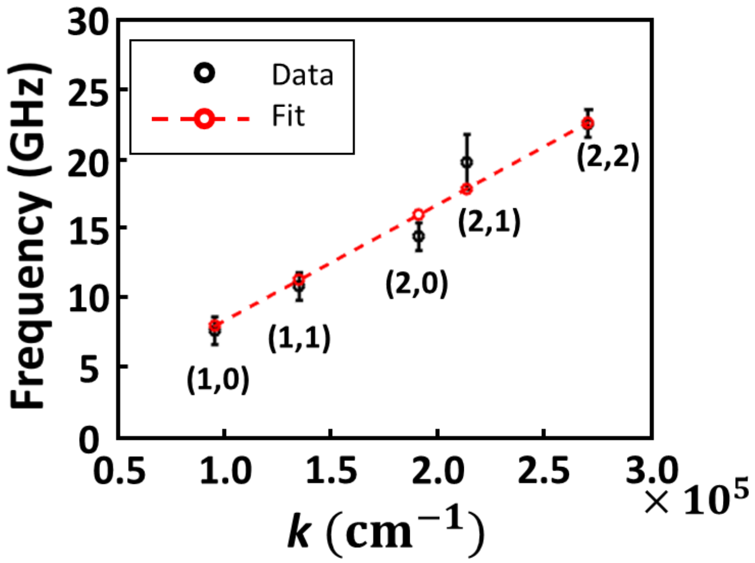

Supplementary Figure 3: Fit of the phonoic modes to Supplementary Equation (4). The error in the frequencies is the FFT resolution obtained from the time duration of each frequency component in the signal.

Magnetic Dynamics

The magnetic dynamics in the absence of damping are given by the well known Landau-Lifshitz (LL) equation

$$\frac{\partial \boldsymbol{m}}{\partial t} = -\gamma \mathbf{m} \times \mathbf{H}_{\mathrm{eff}} \tag{5}$$

Where γ is the gyromagnetic ratio, $= \frac{\mathbf{M}}{M_S}$, M_S is the Saturation Magnetization and $\mathbf{H}_{\text{eff}}$ is the effective field. We neglect the magnetic permeability, μ_0 since $\mu_0 = 1$ in the cgs system. As is customary, a new Cartesian frame of reference is introduced where the 3-axis points along direction of the magnetization vector, the 2-axis is in the film plane, and the 1- axis is orthogonal to the 1 and 2 directions. (Supplementary Fig. 2b) The transformation is given by

$$\begin{pmatrix} m_x \\ m_y \\ m_z \end{pmatrix} = \begin{pmatrix} \cos\theta_{\text{M}}\cos\varphi_{\text{M}} & -\sin\varphi_{\text{M}} & \sin\theta_{\text{M}}\cos\varphi_{\text{M}} \\ \cos\theta_{\text{M}}\sin\varphi_{\text{M}} & \cos\varphi_{\text{M}} & \sin\theta_M\sin\varphi_M \\ -\sin\theta_{\text{M}} & 0 & \cos\theta_{\text{M}} \end{pmatrix} \begin{pmatrix} m_1 \\ m_2 \\ m_3 \end{pmatrix} \tag{6}$$

The effective field is given by

$$\mathbf{H_{eff}} = -\nabla_{\text{m}} G \tag{7}$$

Where $\nabla_{\text{m}} = \left(\frac{\partial}{\partial m_1}, \frac{\partial y}{\partial m_2}, \frac{\partial y}{\partial m_3}\right)$ and G is the free energy of the magnetic system. Assuming an infinite thin film the free energy is given by

$$G = -\mathbf{H}\cdot\mathbf{m} + 2\pi M_S m_z^2 \tag{8}$$

Where M_{S} is the saturation magnetization**.** Assuming the magnetization is pointed along the equilibrium direction and allowing for small deviations in the 1 and 2 directions we can Taylor expand the partial derivatives keeping only the linear terms. This gives the following expression for the effective field

$$\mu_0\mathbf{H_{eff}} = -\begin{pmatrix} G_{11}m_1 + G_{12}m_2 \\ G_{12}m_1 + G_{22}m_2 \\ G_3 \end{pmatrix} \tag{9}$$

Where $G_i = \frac{\partial}{\partial m_i} G \mid_{\boldsymbol{m}=\boldsymbol{m}_0}$ and $G_{ij} = \frac{\partial}{\partial m_i \partial m_j} G \mid_{\boldsymbol{m}=\boldsymbol{m}_0}$. Assuming $m_{1,2} = m_{1,2}^0 e^{i\omega_M t}$ and $m_3 = 1$, the LL equation (Supplementary Eq. 5) is solved which gives the following system of equations

$$\begin{pmatrix} G_{11} - G_3 & \frac{i\omega_M}{\gamma} \\ -\frac{i\omega_M}{\gamma} & -(G_3) \end{pmatrix} \begin{pmatrix} m_1 \\ m_2 \end{pmatrix} = \begin{pmatrix} 0 \\ 0 \end{pmatrix} \tag{10}$$

Setting the Determinant equal to zero gives the well known Kittel formula

$$\left(\frac{\omega_M}{\gamma}\right)^2 = (H\cos(\theta_\mathrm{M} - \theta_\mathrm{H}) - 4\pi M_\mathrm{S}\cos^2\theta_\mathrm{M})(H\cos(\theta_\mathrm{M} - \theta_\mathrm{H}) - 4\pi M_\mathrm{S}\cos 2\theta_\mathrm{M}) \tag{11}$$

Where the angles θ_M and θ_H are the direction of the magnetization and the applied field with respect to the surface normal. (Supplementary Fig. 2) The fitted frequencies are shown in Supplementary Figure 4 and yielded values of $\gamma = 1.98^{+0.02}_{-0.01} \times 10^7$ rad·Oe^{-1}·s^{-1} and $M_\mathrm{S} = 203^{+8}_{-16}$ emu·cm^{-3}.

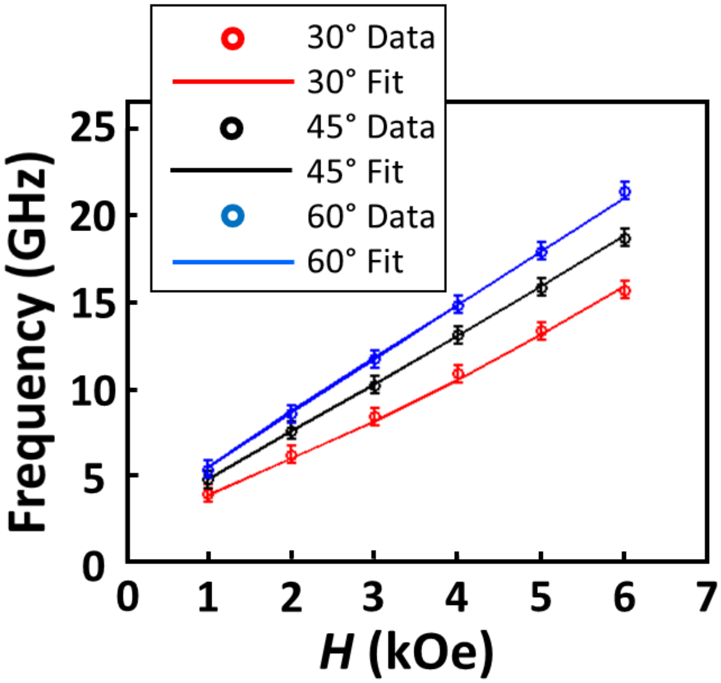

Supplementary Figure 4: Fits of the magnetic resonances to Supplementary Equation (11) at $\theta_H = 30°$, 45° and 60° over a range of applied field strengths. The error bars are given by the 95% confidence intervals from the least squares curve fitting algorithm.

Coupled Dynamics

When magnon-phonon coupling is present there is an added term to the magnetic free energy as well as the elastic energy density. This term is related to the orientation of the magnetization components with respect to the corresponding dynamic strains. The coupling term is given by

$$G^d = b_1\left[\varepsilon_{xx}(x,t)m_x^2 + \varepsilon_{yy}(x,t)m_y^2 + \varepsilon_{zz}(x,t)m_z^2\right] + 2b_2\left[\varepsilon_{xy}(x,t)m_x m_y + \varepsilon_{xz}(x,t)m_x m_z + \varepsilon_{yz}m_y m_z\right] \tag{12}$$

The effective field of the magnetic system is now given by $\mathbf{H_{eff}} = -\nabla_{\mathrm{m}} G^{tot}$ where $G^{tot} = G + G^d$. The magnetization components are assumed to follow the spatial profile of the phononic vibrations, so we assume a plane-wave ansatz of the form $m_i = m_i^0 e^{i(\boldsymbol{k}\cdot\boldsymbol{r}-\omega t)}$. The system of equations is now

$$\begin{pmatrix} G_{11} - G_3 & \frac{i\omega}{\gamma} \\ -\frac{i\omega}{\gamma} & -(G_3) \end{pmatrix}\begin{pmatrix} m_1 \\ m_2 \end{pmatrix} = \begin{pmatrix} -G_1^d \\ -G_2^d \end{pmatrix} \tag{13}$$

Where G_i^d is defined in the same way as G_i. Transforming to the (*1*,*2*,*3*) coordinate system and keeping in mind that the system is two dimensional so that all strain components which have a z-dependence can be neglected gives the following Magneto-Elastic contribution to the magnetization equations of motion.

$$G_1^d = \left(b_1 w_1 i k_x + \frac{1}{2} b_2 w_2 i k_y\right) u_x + \left(b_1 w_3 i k_y + \frac{1}{2} b_2 w_2 i k_x\right) u_y \tag{14}$$

$$G_2^d = \left(-b_1 w_4 i k_x + b_2 w_5 i k_y\right) u_x + \left(b_1 w_4 i k_y + b_2 w_5 i k_x\right) u_y \tag{15}$$

$$w_1 = \sin 2\theta_{\mathrm{M}} \cos^2 \varphi_{\mathrm{mp}}$$

$$w_2 = \sin 2\theta_{\mathrm{M}} \sin 2\varphi_{\mathrm{mp}}$$

$$w_3 = \sin 2\theta_{\mathrm{M}} \sin^2 \varphi_{\mathrm{mp}}$$

$$w_4 = \sin \theta_{\mathrm{M}} \sin 2\varphi_{\mathrm{mp}}$$

$$w_5 = \sin \theta_{\mathrm{M}} \cos 2\varphi_{\mathrm{mp}}$$

Where φ_{mp} is the in-plane angle between the phononic k-vector and the magnetization vector. In two dimensions the magnetic system is driven by both u_x and u_y displacements. b_1 couples the corresponding magnetization component with normal strains and b_2 with shear strains.

Additionally, $M_S G^d$ is added to the elastic free energy (Supplementary Eq. 1). This magneto-elastic energy term modifies the stress tensor (Supplementary Eq. 3) due to the precession of the magnetization. Solving Supplementary Equation (2) with the modified stress tensor gives elastic equations for the displacement which depend on the magnetization components

$$u_x = \frac{i(2M_S b_1 k_x w_1 + 4M_S b_2 k_y w_2)m_1 - i(2M_S b_1 k_x w_4 - 2M_S b_2 k_y w_5)m_2}{\left(C_{xxxx} k_x{}^2 + C_{xxyy} k_x k_y + \frac{C_{xyxy}}{2}(k_y{}^2 + k_x k_y) - \rho\omega^2\right)} \quad (16)$$

$$u_y = \frac{i(2M_S b_1 k_y w_3 + 4M_S b_2 k_x w_2)m_1 + i(2M_S b_1 k_y w_4 + 2M_S b_2 k_x w_5)m_2}{\left(\frac{C_{xyxy}}{2}(k_x k_y + k_x{}^2) + C_{yyyy} k_y{}^2 + C_{xxyy} k_x k_y - \rho\omega^2\right)} \quad (17)$$

Plugging Supplementary Equations (16) and (17) into Supplementary Equations (14) and (15) and combining like terms in Supplementary Equation (13) gives a new system of equations for the precession of the magnetization with only the variables m_1 and m_2. Solving this system of equations by setting the Determinant equal to zero and factoring gives the following

$$(\omega^2 - \omega_{\mathrm{ph}}{}^2)\{(\omega^2 - \omega_{\mathrm{M}}{}^2)(\omega^2 - \omega_{\mathrm{ph}}{}^2) - \omega_{\mathrm{C}}{}^4\} = 0 \quad (18)$$

Where

$$\omega_C{}^4 = \frac{\gamma M_S}{\rho}(\omega_1 C_2 + \omega_2 C_1)k^2$$

$$\omega_1 = \gamma(G_{11} - G_3)$$

$$\omega_2 = \gamma(-G_3)$$

$$C_1 = b_1{}^2 (\cos^2\varphi_k w_1{}^2 + \sin^2\varphi_k w_3{}^2) + \frac{3}{4}b_1 b_2 \sin 2\varphi_k w_2(w_1 + w_3) + \frac{1}{2}b_2{}^2 w_2{}^2$$

$$C_2 = b_1{}^2 w_4{}^2 + 2b_2{}^2 w_5{}^2$$

Supplementary Equation (18) results in three solutions. One of which is the phononic resonance and the other two are attributed to the hybridized magnon-phonon.

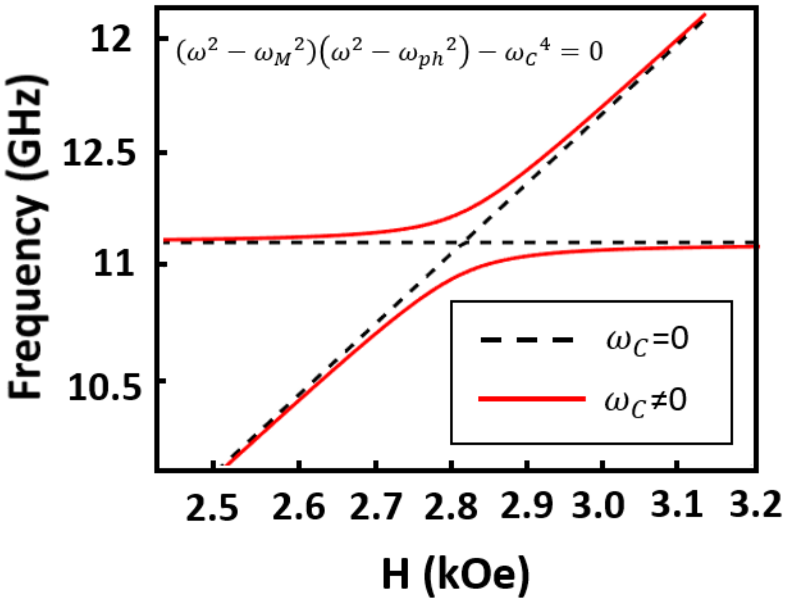

Supplementary Figure 5: Example of solution to the magnon-phonon part of Supplementary Equation (18). When $\omega_C = 0$ the two solutions are attributed ot the phononic and magnonic

resonances. If $\omega_C \neq 0$ then the two systems are coupled and close to the region where the magnon and phonon frequencies are degenerate the avoided crossing produces two solutions which have both magnon and phonon character.

Supplementary References

[1] Dreher, L., Weiler, M., Pernpeintner, M., Huebl, H., Gross, R., Brandt, M. S. & Goennenwein, S.T.B.Elastically Driven Ferromagnetic Resonance in Nickel Thin Films. *Phys. Rev. B.* **86**, 134415 (2012).

[2] Levinson, M. Free Vibrations of a Simply Supported, Rectangular Plate: An Exact Elasticity Solution. *J.Sound Vib.* **98**, 289-298 (1985).

[3] Wang, D., Liu, W. & Zhang, H. Superconvergent isogeometric free vibration analysis of Euler-Bernoulli beams and Kirchoff plates with new higher order mass matrices. *Comput. Methods Appl. Mech. Engrg.* **286**, 230–267 (2015).

[4] Lautrup B. *Physics of Continuous Matter: Exotic and Everyday Phenomena in the Macroscopic World.* (CRC, 2011).

[5] Ledbetter, H. M. & Reed, R. P. Elastic Properties of Metals and Alloys, I. Iron, Nickel, and Iron-Nickel Alloys. *J. Phys. Chem. Ref. Data.* **2**, 531 (1973).
